# Deliberate self‐harm in adolescents with OTC‐related psychiatric disorders: A study of prevalence and associated factors

**DOI:** 10.1002/pcn5.70271

**Published:** 2025-12-15

**Authors:** Toshihiko Matsumoto, Takashi Usami, Akiho Nishimura, Sayako Higuchi, Kyoji Okita, Takuya Shimane

**Affiliations:** ^1^ Department of Drug Dependence Research National Center of Neurology and Psychiatry, National Institute of Mental Health Kodaira Tokyo Japan; ^2^ Department of Psychiatry Center Hospital, National Center of Neurology and Psychiatry Kodaira Japan; ^3^ Fukuoka Prefectural Psychiatric Center Dazaifu Hospital Dazaifu Tokyo Japan; ^4^ Department of Forensic Psychiatry Center Hospital, National Center of Neurology and Psychiatry Kodaira Japan

**Keywords:** 3,4‐Methylenedioxyemthamphetamine, adolescents, deliberate self‐harm, over‐the‐counter drugs, substance use disorder, suicide

## Abstract

**Aim:**

This study examines the issues of over‐the‐counter (OTC) drug abuse among adolescents from the perspective of deliberate self‐harm and suicide. It aims to elucidate the clinical characteristics of psychiatric disorders related to OTC drug use and explore treatment approaches in the context of self‐harm and suicide prevention.

**Methods:**

The study analyzed 93 adolescent cases (ages 10–19) of psychiatric disorders primarily attributed to OTC drug use, extracted from the “2024 Nationwide Survey on Drug‐Related Psychiatric Disorders in Psychiatric Medical Facilities in Japan.” Cases were categorized into two groups based on the presence or absence of incidents of deliberate self‐harm within the past year. Clinical variables were compared between the two groups.

**Results:**

Incidents of deliberate self‐harm within the past year were observed in 82.8% of the cases. Clinical features significantly associated with deliberate self‐harm included being female, having an educational level of high school dropout or lower (including current enrollment in middle or high school), and engaging in sub‐threshold drug use not meeting the diagnostic criteria for addiction. Furthermore, recent OTC drug use, severity of substance use disorder, and the presence of comorbid psychiatric disorders were not associated with “deliberate self‐harm” among adolescents with OTC‐related psychiatric disorders. The use of existing medical or non‐medical resources designed for drug addiction treatment was also not associated with self‐harming behavior.

**Conclusion:**

From the standpoint of suicide and self‐harm prevention, conventional approaches centered on substance use disorder treatment and recovery support may not be sufficiently effective for this population.

## INTRODUCTION

In recent years, the abuse of over‐the‐counter (OTC) drugs has become a significant concern in the clinical practice of substance use disorders. The biennial “Nationwide Psychiatric Hospital Survey on Drug‐Related Mental Disorders (NPH survey),” conducted by our research team, indicates a steady increase in psychiatric disorders primarily associated with OTC drug abuse since 2018.^1^

This trend has been particularly pronounced among adolescents (ages 10–19). In the 2014 survey, conducted at the peak of the new psychoactive substances (NPS) abuse epidemic, nearly half of the cases of drug‐related psychiatric disorders among adolescents identified NPS as their primary substance of abuse. However, with the withdrawal of stores selling NPS due to stricter regulations and the revision of the Pharmaceutical Affairs Law, the 2016 survey showed a sharp decline in the number of drug‐related psychiatric disorders among adolescents, reaching zero in 2018. Yet, coinciding with the disappearance of NPS, cases of OTC drug‐related psychiatric disorders among adolescents began to emerge in the 2016 survey and have steadily increased with each subsequent survey. A recent survey conducted in 2024 revealed that the number of teenage patients diagnosed with drug‐related mental disorders was more than 5 times higher than in 2014—when NPS abuse peaked—and over 10 times higher than in 2016—when NPS abuse had declined. Among these patients, 71.5% primarily abused OTC medications.^1^

It is important to emphasize that adolescents did not simply switch to OTC drug abuse because NPS became unavailable. Our comparative analysis of cases with NPS‐related psychiatric disorders among adolescents in the 2014 survey and cases with OTC drug‐related psychiatric disorders among adolescents in the 2018 survey revealed stark differences in their backgrounds.^2^ Specifically, the former group was predominantly male, often had discontinued education early, and exhibited pronounced tendencies toward delinquency and criminal behavior. This group matches the “classic” image of patients using illicit drugs such as methamphetamine in Japan.^3^ In contrast, the latter group consisted primarily of females, many of whom were still enrolled in middle or high school and did not exhibit delinquent or criminal behavior.

This suggests that easier access to OTC drugs for children, by whatever reason, may have created a new group of drug abusers. Furthermore, treating these individuals within the conventional framework of substance use disorder treatment is often highly challenging. This difficulty arises because many of these patients present with multiple comorbid psychiatric disorders and, more critically, frequently engage in deliberate self‐harm or suicide attempts.^4^ As a result, treatment efforts must often prioritize safety and suicide prevention over abstinence.

Meanwhile, Japan is also facing another serious social issue: a rise in suicide rates among school‐aged children and adolescents. Although the suicide rate among this demographic had been gradually increasing over the past two decades, it surged sharply during the COVID‐19 pandemic and has continued to rise even after the pandemic ended. Notably, the most significant increase has been observed among female high school students, whose suicide rate rose by a factor of 2.3 between 2019 and 2024.^5^ Generally, a gender paradox exists between completed and attempted suicides, with males tending to complete suicide more often and females exhibiting more attempts.^6^ However, among Japanese high school students, this pattern is reversed: Female students outnumber their male counterparts in both suicide attempts and completions.

Based on these trends, we have long suspected that the population of adolescent females who abuse OTC drugs—frequently encountered in clinical settings for drug addiction—may significantly overlap with adolescents currently at high risk for suicide. To the best of our knowledge, however, no empirical studies have yet confirmed a causal relationship between these phenomena. In this context, Tachikawa conducted a correlation analysis using macro‐level data from the Ministry of Health, Labour and Welfare, the Ministry of Education, Culture, Sports, Science and Technology, and the Ministry of Economy, Trade and Industry. His analysis revealed an intriguing finding: A moderate correlation, with a coefficient of 0.780, exists between the growth rate of domestic OTC drug sales and the increase in suicide rates among teenage girls.^7^ Furthermore, our daily clinical experience consistently involves managing self‐destructive behaviors—such as self‐injury and suicide attempts—among young female patients who abuse OTC drugs, often under urgent and challenging circumstances.

Therefore, this study aimed to examine the issue of OTC drug abuse among adolescents from the perspective of deliberate self‐harm and suicide. Specifically, it sought to elucidate the clinical characteristics of psychiatric disorders related to OTC drug use and to explore treatment approaches for these cases from the perspective of self‐harm and suicide prevention. This was done using data from the NPH survey.

## METHODS

### NPH survey

#### Overview

First, we would like to provide an overview of the NPH survey, which forms the basis of the present study. The NPH survey has been conducted almost biennially since 1987 by the Department of Drug Dependence Research, National Institute of Mental Health, National Center of Neurology and Psychiatry, targeting inpatient psychiatric facilities across Japan.^1^ It is the country's only complete enumeration survey focusing on patients with drug‐related psychiatric disorders.

Each survey targets a 2‐month period, specifically September and October of the survey year. Eligible subjects include patients who received inpatient or outpatient treatment at the participating facilities and were diagnosed with disorders classified under ICD‐10 F1 categories (mental and behavioral disorders due to psychoactive substance use) arising from substances other than alcohol. While patients who use alcohol alone are excluded, those who use alcohol in combination with other substances are included. Data collection is performed by designated psychiatrists at each facility, who extract and transcribe relevant information on patients with drug‐related psychiatric disorders from medical records onto survey forms. During the survey period, announcements are made at the participating medical institutions, and patients who meet the criteria are given the opportunity to opt out.

In the 2024 survey, 1098 (72.0%) of the 1525 eligible facilities cooperated in the survey, and the survey was conducted from September to October 2024, with a total of 2702 drug‐related psychiatric disorder cases collected during the survey period.

#### Survey items

The survey consistently collects data using identical questionnaires and definitions across different years. The survey items are as follows: age group, biological sex, educational background, employment status, criminal history, current alcohol‐related problems (at a level corresponding to an ICD‐10 F1 diagnosis), drug use history within the past year, current primary substance, ICD‐10 F1 subcategory diagnosis, and presence of comorbid psychiatric disorders (classified under ICD‐10 broad categories).

The term primary substance refers to the psychoactive substance considered to be most clinically associated with the patient's current psychiatric symptoms. Attending psychiatrists selected one substance from the following categories: methamphetamine, volatile solvents (e.g., toluene, paint thinner), cannabis, cocaine, heroin, 3,4‐Methylenedioxymethamphetamine (MDMA), hallucinogens other than MDMA, NPS, prescribed hypnotics or anti‐anxiety medications (primarily benzodiazepine receptor agonists), analgesics (prescribed non‐opioid), analgesics (prescribed opioid), OTC drugs (e.g., cough medicines, cold remedies, analgesics, and hypnotics), attention deficit hyperactivity disorder medications, others, or polymedication. When multiple substances were judged to have contributed equally to the psychiatric condition, polymedication was selected.

#### New items in the 2024 survey

The first item relates to the classification of abused OTC drugs. Information regarding the specific OTC products most frequently abused was collected using an independently developed classification based on the primary active ingredient (multiple selections were allowed). The seven categories were as follows:

● Codeine‐containing group (COD group): cough and cold remedies containing codeine or dihydrocodeine phosphate.
● Dextromethorphan‐based group (DXM group).
● Bromvalerylurea‐based group (BVU group).
● Diphenhydramine‐based group (DPH group).
● Allylisopropylacetylurea‐based group (AIU group).
● Caffeine monotherapy group (CAF group).
● Other OTC drug groups (other group).

The second item pertains to incidents of deliberate self‐harm within the past year, for which method‐specific data were not collected. This category includes a broad range of behaviors, such as non‐suicidal self‐harm (e.g., self‐cutting), intentional overdose of medication as a coping mechanism for emotional distress, and self‐destructive acts driven by clear suicidal intent. Notably, a systematic review has demonstrated that deliberate self‐harm is a significant predictor of long‐term suicide mortality.^8^

Finally, an item was included regarding the utilization of both medical and non‐medical resources for drug addiction treatment. Specifically, the resources commonly employed in the treatment and recovery support for drug addiction in Japan were examined. The medical resources included two aspects: experience of participation in outpatient group therapy for addiction (such as SMARPP [Serigaya Methamphetamine Relapse Prevention Program]) and experience of psychiatric hospitalization due to drug‐related issues. The non‐medical resources also included two aspects: experience of participation in self‐help groups for drug addiction, such as Narcotics Anonymous, and experience in utilizing private addiction recovery facilities, such as the Drug Addiction Rehabilitation Center (DARC).

### Study subjects and methods

#### Study subjects

The study consisted of adolescent cases (ages 10–19) with psychiatric disorders primarily attributed to OTC drug use, extracted from a total of 2702 drug‐related psychiatric cases collected in the 2024 NPH survey.

#### Variables

For these subjects, data were extracted from the NPH survey database for analysis. The following variables were included: educational background, current employment status, criminal history, details of OTC drug abuse (classification of abused OTC drugs and whether OTC drug abuse occurred in the past year), use of other psychoactive substances (current alcohol‐related issues, history of illegal drug use, and history of prescription medication abuse), subcategory diagnosis under the ICD‐10 F1 classification (“Mental and behavioral disorders due to psychoactive substance use”), co‐occurring psychiatric disorders (ICD‐10 major categories), experience utilizing medical or non‐medical resources for drug addiction (outpatient group therapy for addiction, psychiatric hospitalization, self‐help groups, and private addiction recovery facilities), and information regarding incidents of “deliberate self‐harm” in the past year.

#### Statistical analysis

Adolescent cases of OTC drug‐related psychiatric disorders (hereafter referred to as “OTC cases”) were divided into two groups based on the presence or absence of incidents of “deliberate self‐harm” in the past year. Pearson's *χ* ^2^ test was used to compare the two groups on the aforementioned survey variables. However, for cells with an expected value less than 5, Fisher's exact test was also performed. Note that this study is exploratory in nature, and no adjustments for multiple comparisons were made.

Statistical analysis was performed using IBM spss version Statistics 29, with a two‐tailed significance level set at 5%.

### Ethical considerations

This study was conducted with the approval of the Ethics Committee of the National Center of Neurology and Psychiatry (Approval number: A2024‐041).

## RESULTS

Among the 2702 cases of drug‐related psychiatric disorders collected in the 2024 NPH survey, 130 cases involved adolescents. Of these, 93 cases where the primary substance used was OTC drugs were included in the present study.

Table 1 summarizes the profiles of these 93 subjects. The biological sex distribution was overwhelmingly female, with 10 males (10.8%) and 83 females (89.2%). In terms of educational background, the majority were currently enrolled in middle or high school (69 cases, 74.2%), while a smaller proportion had an education level of high school dropout or below (12 cases, 12.9%) or had already graduated from high school or higher (12 cases, 12.9%). Few subjects had a criminal history: three (3.2%) had a history of arrest for drug‐related offenses, and five (5.4%) had been arrested for non‐drug–related crimes. None had been placed in correctional facilities such as juvenile classification homes or juvenile detention centers.

**Table 1:** Case profile of over‐the‐counter (OTC) drug‐related psychiatric disorders in adolescents.

			Adolescent cases of OTC drug‐related psychiatric disorders	
			*N* = 93	
			*N*	%
Biological sex	Male		10	10.8
	Female		83	89.2
Educational background	Currently enrolled in middle or high school		69	74.2
	Educational attainment of high school dropout or less		12	12.9
	Educational attainment of high school diploma or higher		12	12.9
Currently employed			9	9.7
Criminal history	History of arrest for drug‐related offenses		3	3.2
	History of arrests for non‐drug–related offenses		5	5.4
	History of incarceration in a correctional facility		0	0.0
Details of OTC drug use	Classification of OTC drugs of abuse (multiple responses)	COD group (codeine‐containing group)	48	51.6
		DXM group (dextromethorphan‐based group)	55	59.1
		BVU group (bromvalerylurea‐based group)	10	10.8
		AIU group (allylisopropylacetylurea‐based group)	4	4.3
		DPH group (diphenhydramine‐based group)	16	17.2
		CAF (caffeine) monotherapy group	3	3.2
		Other group (other OTC drugs)	2	2.2
	OTC drug abuse within the past year		82	88.2
Issues related to other psychoactive substances	Current alcohol‐related issues		11	11.8
	Experience with illegal drug use		3	3.2
	Experience with prescription drug abuse		16	17.2
Diagnosis related to drug use (ICD‐10 classification F1 sub‐diagnosis)	F1x.0	Acute intoxication	20	21.5
	F1x.1	Harmful use	59	63.4
	F1x. 2	Dependence syndrome	36	38.7
	F1x.3	Withdrawal state	3	3.2
	F1x.4	Withdrawal state with delirium	3	3.2
	F1x. 5	Psychotic disorder	3	3.2
	F1x. 6	Amnesic syndrome	0	0.0
	F1x. 7	Residual and late‐onset psychotic disorders	1	1.1
	F1x. 8	Other mental and behavioral disorders	0	0.0
Comorbid psychiatric disorders	F0	Mental disorders due to known physiological conditions (including symptomatic organic mental disorders)	1	1.1
	F2	Schizophrenia, schizotypal, and delusional disorders	3	3.2
	F3	Mood (affective) disorders	23	24.7
	F4	Neurotic, stress‐related, and somatoform disorders	46	49.5
	F5	Behavioral syndromes associated with physiological disturbances and physical factors	4	4.3
	F6	Disorders of adult personality and behavior	9	9.7
	F7	Intellectual disabilities	6	6.5
	F8	Disorders of psychological development	25	26.9
	F9	Behavioral and emotional disorders with onset usually occurring in childhood and adolescence	20	21.5
Experience in utilizing resources for treatment and recovery	Experience of participation in outpatient group therapy for addiction		12	12.9
	Experience of psychiatric hospitalization due to drug‐related issues		44	47.3
	Experience of participation in self‐help groups for drug addiction		4	4.3
	Experience in utilizing private addiction recovery facilities		0	0.0
Incident of “deliberate self‐harm” within the past year			77	82.8

Regarding the types of OTC drugs abused, the most frequently used was the DXM group (55 cases, 59.1%), closely followed by the COD group (48 cases, 51.6%). These two categories were markedly more prevalent than others, followed by the DPH group (16 cases, 17.2%) and the BVU group (10 cases, 10.8%). Recent (past‐year) OTC drug abuse was observed in 82 subjects (88.2%), indicating ongoing substance use in the majority of the subjects. Regarding the use of other psychoactive substances, prescription drug abuse was the most common (16 cases, 17.2%), followed by alcohol‐related issues (11 cases, 11.8%). Experience with illegal drug use was rare (3 cases, 3.2%).

With regard to ICD‐10 diagnoses, the majority of subjects were classified under harmful use (59 cases, 63.4%) within the F1 subcategories reflecting substance use‐related states. Fewer subjects met the criteria for dependence syndrome, while acute intoxication (20 cases, 21.5%) was the next most frequent diagnosis. Diagnoses of withdrawal state (3 cases, 3.2%) and psychotic disorder (3 cases, 3.2%) were rare. The most common comorbid psychiatric disorders were F4 Neurotic, stress‐related, and somatoform disorders (46 cases, 49.5%), followed by F8 Disorders of psychological development (25 cases, 26.9%), F3 Mood (affective) disorders (23 cases, 24.7%), and F9 Behavioral and emotional disorders with onset usually occurring in childhood and adolescence (20 cases, 21.5%).

In terms of resources utilized for treatment and recovery, nearly half of the subjects had experienced psychiatric hospitalization (44 cases, 47.3%). However, relatively few had participated in outpatient group therapy for addiction (12 cases, 12.9%). Meanwhile, the use of non‐medical resources was extremely low: Participation in self‐help groups was reported by only 4 subjects (4.3%), and no subjects had utilized private addiction recovery facilities.

Regarding deliberate self‐harm within the past year, 77 of the 93 subjects (82.8%) were identified as having engaged in such behavior.

Table 2 presents the results of comparisons between subjects with and without a past‐year incident of deliberate self‐harm. The analysis revealed that factors significantly associated with incidents of deliberate self‐harm included being female (female rate: 85.5% vs. 14.5%, *p* < 0.05), having an educational background of currently being enrolled in middle or high school or having a high school dropout or lower status (90.9% vs. 68.8%, *p* < 0.05), and being diagnosed with harmful use under the ICD‐10 F1 subcategory diagnosis (68.8% vs. 37.5%, *p* < 0.05). In contrast, no significant associations were observed between the presence or absence of incidents of deliberate self‐harm and the type of abused OTC drug, the presence of OTC drug abuse within the past year, the diagnosis of dependence syndrome under ICD‐10 F1, the presence of various comorbid psychiatric disorders, or the utilization of medical or non‐medical resources.

**Table 2:** Comparison of presence and absence of deliberate self‐harm in adolescents with over‐the‐counter (OTC)‐related psychiatric disorder.

			Comparison of presence and absence of deliberate self‐harm within the past year					
			Presence of deliberate self‐harm		Absence of deliberate self‐harm		*χ* ^2^ value	*p* *
			*N* = 77		*N* = 16			
			*N*	%	*N*	%		
Biological sex	Male		6	60.0	4	40.0	4.088	0.043 (0.044)**
	Female		71	85.5	12	14.5		
Educational background	Educational background of currently being enrolled in middle or high school, or educational attainment of high school dropout or less		70	90.9	11	68.8	5.709	0.016 (0.030)**
	High school graduate or higher		7	9.1	5	31.3		
Currently employed			6	7.9	3	20.0	2.060	0.151 (0.164)
Criminal history	History of arrest for drug‐related offenses		3	3.9	0	0.0	0.644	0.422 (0.564)
	History of arrests for non‐drug–related offenses		5	6.5	0	0.0	1.099	0.295 (0.380)
	History of incarceration in a correctional facility		0	0.0	0	0.0	ー	ー
Details of OTC drug use	Classification of OTC drugs of abuse (multiple responses)	COD group (codeine‐containing group)	41	53.9	7	43.8	0.551	0.458 (0.320)
		DXM group (dextromethorphan‐based group)	44	57.9	11	68.8	0.648	0.421 (0.304)
		BVU group (bromvalerylurea‐based group)	9	11.8	1	6.3	0.427	0.514 (0.449)
		AIU group (allylisopropylacetylurea‐based group)	2	2.6	2	12.5	3.095	0.079 (0.138)
		DPH group (diphenhydramine‐based group)	13	17.1	3	18.8	0.025	0.875 (0.560)
		CAF (caffeine) monotherapy group	3	3.9	0	0.0	0.653	0.419 (0.560)
		Other group (other OTC drugs)	2	2.6	0	0.0	0.430	0.512 (0.681)
	OTC drug abuse within the past year		69	89.6	13	81.3	0.888	0.346 (0.285)
Issues related to other psychoactive substances	Current alcohol‐related issues		10	13.0	1	9.1	0.577	0.448 (0.398)
	Experience with illegal drug use		2	2.6	1	6.3	0.566	0.452 (0.436)
	Experience with prescription drug abuse		14	18.2	2	12.5	0.300	0.584 (0.449)
Diagnosis related to drug use (ICD‐10 classification F1 sub‐diagnosis)	F1x.0	Acute intoxication	16	20.8	4	25.0	0.140	0.708 (0.467)
	F1x.1	Harmful use	53	68.8	6	37.5	5.607	0.018 (0.020)**
	F1x. 2	Dependence syndrome	28	36.4	8	50.0	1.038	0.308 (0.229)
	F1x.3	Withdrawal state	3	3.9	0	0.0	0.644	0.422 (0.564)
	F1x.4	Withdrawal state with delirium	2	2.6	1	6.3	0.566	0.452 (0.436)
	F1x. 5	Psychotic disorder	3	3.9	0	0.0	0.644	0.422 (0.564)
	F1x. 6	Amnesic syndrome	0	0.0	0	0.0	ー	ー
	F1x. 7	Residual and late‐onset psychotic disorders	1	1.3	0	0.0	0.210	0.647 (0.828)
	F1x. 8	Other mental and behavioral disorders	0	0.0	0	0.0	ー	ー
Comorbid psychiatric disorders	F0	Mental disorders due to known physiological conditions (including symptomatic organic mental disorders)	1	1.3	0	0.0	0.210	0.647 (0.828)
	F2	Schizophrenia, schizotypal, and delusional disorders	2	2.6	1	6.3	0.566	0.452 (0.436)
	F3	Mood (affective) disorders	19	24.7	4	25.0	0.001	0.978 (0.601)
	F4	Neurotic, stress‐related, and somatoform disorders	41	53.2	5	31.3	2.564	0.109 (0.092)
	F5	Behavioral syndromes associated with physiological disturbances and physical factors	3	3.9	1	6.3	0.178	0.673 (0.537)
	F6	Disorders of adult personality and behavior	9	11.7	0	0.0	2.071	0.150 (0.168)
	F7	Intellectual disabilities	6	7.8	0	0.0	1.333	0.248 (0.311)
	F8	Disorders of psychological development	22	28.6	3	13.8	0.650	0.420 (0.320)
	F9	Behavioral and emotional disorders with onset usually occurring in childhood and adolescence	17	22.1	3	18.8	0.087	0.768 (0.533)
Experience in utilizing resources for treatment and recovery	Experience of participation in outpatient group therapy for addiction		8	10.4	4	25.0	2.516	0.113 (0.122)
	Experience of psychiatric hospitalization due to drug‐related issues		36	46.8	8	50.0	0.247	0.884
	Experience of participation in self‐help groups for drug addiction		3	3.9	1	6.3	0.178	0.673 (0.537)
	Experience in utilizing private addiction recovery facilities		0	0.0	0	0.0	ー	ー

* The significance level was set at 5%, and the numbers in parentheses in the *p*‐value column are the results of Fisher's exact test.

** *p* < 0.05.

Among the variables that demonstrated significant differences between the two groups, biological sex and educational background were considered potential confounders. Consequently, a logistic regression analysis was conducted, with deliberate self‐harm within the past year serving as the dependent variable, and gender and educational background as the independent variables (Table 3). Given the small sample size, to avoid overfitting due to excessive variable inclusion, only variables with a high potential for confounding among those showing significant differences in the bivariate analysis were selected as independent variables.

**Table 3:** Logistic regression analysis was conducted, with deliberate self‐harm within the past year.

	*β*	df	*p*	AOR	95% CI	
					Lower	Upper
Female	1.332	1	0.075	3.789	0.876	16.391
Educational background of currently being enrolled in middle or high school, or educational attainment of high school dropout or less	1.483	1	0.031	4.407	1.143	16.986

Abbreviations: AOR, adjusted odds ratio; CI, confidence interval.

The findings indicated that educational background emerged as the sole factor demonstrating a statistically significant correlation with recent deliberate self‐harm. Specifically, the odds of reporting deliberate self‐harm in the past year increased by more than four times for individuals currently enrolled in middle or high school or having a high school dropout or lower status (*p* = 0.031, adjusted odds ratio 4.407 [95% confidence interval: 1.143–16.986]).

## DISCUSSION

The present study represents the first investigation in Japan to examine the issue of OTC drug abuse among adolescents—an issue that has recently become a major social concern—from the perspectives of both substance use disorder and self‐harm/suicide.

The most significant finding of this study is that 82.8% of adolescent patients abusing OTC drugs experienced an incident of deliberate self‐harm within the past year. For reference, among all 2702 cases of drug‐related psychiatric disorders identified in the 2024 NPH survey, the prevalence of deliberate self‐harm in the past year was 19.4%.^1^ It should be emphasized that the NPH survey did not collect detailed information on the specific method of deliberate self‐harm. While the survey item encompassed behaviors such as cutting, intentional overdose, and suicide attempts, these were not differentiated. Therefore, the extremely high prevalence of DSH observed in this study may partly reflect cases where intentional overdose of OTC medications was classified as self‐harm. This limitation warrants caution in interpreting the magnitude of the association.

Furthermore, the absence of a significant association between recent OTC use and deliberate self‐harm should not be interpreted as evidence that OTC use is unrelated to self‐harm risk. Given the cross‐sectional nature of the data, this finding may reflect that self‐harm behaviors persist even after cessation of OTC use or occur in contexts where psychosocial distress—rather than substance use per se—is the primary driver. Future longitudinal studies are needed to clarify these temporal and causal relationships.

The findings of this study are striking for professionals involved in addiction treatment and recovery support. This is due to the fact that the variables associated with incidents of deliberate self‐harm in the past year were limited to sex, educational status (i.e., dropping out of high school or being currently enrolled in middle or high school), and harmful use—a pattern of substance use that falls short of dependence. Furthermore, neither the presence of comorbid psychiatric disorders nor the recent use of OTC drugs was associated with self‐harm or suicide incidents. Similarly, the use of medical and non‐medical resources specialized in drug addiction treatment and recovery showed no significant relationship. These results suggest that, at least from the standpoint of self‐harm and suicide prevention, conventional addiction treatment may not necessarily be an effective intervention for adolescent OTC drug abuse cases.

However, it is crucial to emphasize that this does not imply that psychiatric care is ineffective overall. Indeed, nearly half of our subjects had experienced psychiatric hospitalization, indicating the existence of certain medical needs. It is plausible to assume that complex circumstances underlay these hospitalizations. For some, early psychiatric admission may have helped prevent deliberate self‐harm; for others, psychiatric hospitalization may have occurred as a consequence of self‐harm incidents. If the former were predominant, a negative association between psychiatric hospitalization and self‐harm would have been observed; if the latter were predominant, a positive association would be expected. Given the realities of clinical practice, it is likely that both patterns existed in roughly equal measure, offsetting one another and resulting in a lack of significant association.

More noteworthy, perhaps, is the fact that nearly half of the patients who had been hospitalized were not subsequently referred to specialized addiction recovery resources, including outpatient group therapy for addiction, self‐help groups, and private addiction recovery facilities. There are two possible explanations for this finding. First, the psychiatric hospitals where patients were admitted may not have had programs such as outpatient group therapy for addiction, or attending psychiatrists may have lacked information about addiction‐specific resources and thus did not consider them among the treatment options. Second, attending psychiatrists may have judged that traditional addiction recovery resources were not a good fit for adolescents abusing OTC drugs and therefore refrained from strongly recommending them. While the results of this study cannot conclusively determine which explanation is more valid, we believe the latter possibility is more likely. The rationale is clear. Japan's addiction treatment and recovery infrastructure has historically been developed primarily to address methamphetamine abuse—a long‐standing national issue^3^—and most individuals utilizing these services are middle‐aged men with methamphetamine‐related issues. Such environments are unlikely to provide a safe or supportive space for adolescent females grappling with OTC drug abuse.

Moreover, the philosophy underpinning conventional addiction treatment and recovery may not align with the needs of adolescent OTC drug abuse cases. It is particularly significant that the present study found those at highest risk for deliberate self‐harm were not those with dependence syndrome, but rather those at the level of harmful use—below the threshold for dependence. This is not unexpected. Even in the absence of tolerance acquisition and with a low‐frequency, intermittent pattern of use, overdosing on drugs with self‐destructive intent can be considered deliberate self‐harm. On the other hand, it should be noted that deliberate self‐harm may include methods other than overdosing on OTC drugs. As clearly shown in Table 2, our findings identified a substantial number of individuals who engaged in deliberate self‐harm even during more than a year of cessation of OTC drug abuse, as well as individuals who did not engage in self‐harm despite abusing OTC drugs within the past year. In short, the conventional stance for addiction recovery resources that defines abstinence as the primary goal may not be effective, at least with regard to preventing self‐harm and suicide. Support for adolescents abusing OTC drugs may need to be based on alternative approaches, such as incorporating harm reduction principles into individualized support strategies.^9^

From the perspective of support within daily living environments rather than specialized institutions, our finding regarding the association between educational status and deliberate self‐harm may warrant attention. In the present study, recent deliberate self‐harm was more prevalent among those with an educational attainment of high school dropout or less, as well as among those currently enrolled in middle or high school; conversely, it was less prevalent among those with a high school diploma or higher. This result may reflect the fact that early disengagement from education is associated with lower academic and social skills, which may in turn facilitate self‐harm and suicide, while possessing sufficient skills to complete high school serves a protective role, because Park and Song suggested a potential protective effect of better academic achievement against suicide.^10^ However, considering that 74.2% of the subjects were still enrolled in school, this interpretation remains insufficient. The findings of this study may reflect the critical importance of addressing OTC drug abuse within educational settings and strengthening collaboration between psychiatric services and educational institutions. Indeed, Nott et al. indicated that many of adolescent abusing pharmaceuticals struggle with academic and career concerns, peer conflict, and bullying, leading to escalating substance abuse.^11^ Franke et al. also suggested that adolescents sometimes become isolated in their classrooms or are marginalized by teachers due to their drug abuse.^12^ Based on these findings, it may be an urgent task for educational institutions to implement appropriate measures to address the problem of students' misuse of OTC drugs. Additionally, it may be necessary to formulate strategies to support their academic performance.

There is one last supplementary point we would like to make here. The present study also showed that the most frequently abused OTC drug among the subjects was the DXM group. In the 2024 NPH survey, the most chosen OTC drug among all ages was the COD group, followed by the DXM group.^1^ The present study revealed that when limited to teenagers, this order was reversed. We have already pointed out from the comparison between the 2018 and 2022 NPH survey data that although the COD group remains the most chosen in both surveys,^13^ there has been a significant increase in the number of OTC drug‐related disorder cases choosing DXM in recent years. The present study revealed that this trend has further accelerated, especially among young people, with approximately 60% of teenage OTC drug‐related mental disorder cases choosing the DXM group. Since 2014, the Ministry of Health, Labour and Welfare has imposed sales quantity restrictions on several OTC drugs as “drugs with potential for abuse,” and the COD group has been subject to these restrictions early on. However, despite the fact that a Dextromethorphan single‐ingredient cough suppressant has been abused since its market launch in 2021, and the number of abusers has been increasing, especially among young people, the Ministry of Health, Labour and Welfare has not yet subjected the DXM group to sales quantity restrictions. In this sense, it cannot be denied that this “OTC drug crisis” in Japan has an aspect of a man‐made disaster due to the delay in government measures.

Finally, the limitations of this study must be acknowledged. Although various limitations exist, six major ones are particularly significant. First, the NPH survey did not collect detailed information on the specific method of deliberate self‐harm. While the survey item encompassed behaviors such as cutting, intentional overdose, and suicide attempts, it did not differentiate among these methods. Consequently, we cannot determine the extent to which intentional overdose of OTC medications contributed to the high prevalence of DSH observed in this study. This limitation should be considered when interpreting the findings. Second, the cross‐sectional design precludes any inference of causality between OTC drug use and deliberate self‐harm. The lack of association between recent OTC use and DSH does not imply that OTC use is unrelated to self‐harm risk; rather, it may reflect that self‐harm behaviors persist independently of current substance use or occur in contexts driven by psychosocial distress. Future longitudinal studies are needed to clarify these relationships.

Third, the validity of the collected information has limitations. Because the NPH survey that forms the basis of this study relied on retrospective data extracted from medical records by attending psychiatrists at each facility, inconsistencies in evaluation and judgment criteria among attending psychiatrists are inevitable, and the retrospective nature of the data collection imposes limits on the comprehensiveness of available variables. Fourth, this study, based on cross‐sectional data collection, captures only the associations between deliberate self‐harm and various factors and does not establish causal relationships. Fifth, given the limited sample size and the skewed incidence of “deliberate self‐harm,” the confidence interval for the adjusted odds ratio derived from the logistic regression analysis is extremely wide, indicating substantial uncertainty. Consequently, our findings should be interpreted with caution. Finally, these results reflect conditions within psychiatric settings and should not be generalized to other contexts, such as emergency medicine or educational environments.

Despite these limitations, this study represents the first investigation in Japan to examine the issue of OTC drug abuse among adolescents from the dual perspectives of addiction and self‐harm/suicide. It is expected to make an important contribution to the prevention of youth suicide, which has become a major social concern.

## CONCLUSIONS

This study aimed to examine the issue of OTC drug abuse among adolescents through the lens of deliberate self‐harm and suicide. Using data from the 2024 NPH survey, we sought to elucidate the clinical characteristics of psychiatric disorders related to OTC drug abuse and to explore insights for treatment and recovery support within the context of self‐harm and suicide prevention. As a result, incidents of deliberate self‐harm within the past year were observed in 82.8% of adolescent cases involving OTC drug‐related psychiatric disorders. Clinical features significantly associated with deliberate self‐harm included being female, having an educational level of high school dropout or lower (including current enrollment in middle or high school), and engaging in sub‐threshold drug use not meeting the diagnostic criteria for addiction. On the other hand, recent OTC drug use, severity of dependence, and the presence of comorbid psychiatric disorders were not associated with incidents of deliberate self‐harm. Furthermore, the use of existing medical or non‐medical resources designed for drug addiction treatment did not appear to influence self‐harming behavior. From a suicide and self‐harm prevention standpoint, it was suggested that conventional approaches centered on substance use disorder treatment and recovery support may not be sufficiently effective for adolescents.

Our findings highlight the critical need to integrate suicide prevention strategies into clinical care for adolescents presenting psychiatric disorders related to OTC drug misuse. Moving forward, it is essential to develop a comprehensive support system grounded in harm reduction principles for students engaging in OTC drug misuse, as part of a collaborative suicide prevention strategy between psychiatric services and educational institutions.

## AUTHOR CONTRIBUTIONS

Toshihiko Matsumoto designed this research, and Akiho Nishimura, Sayako Higuchi, and Kyoji Okita collected the data. Toshihiko Matsumoto and Takashi Usami established the participant database and performed the statistical analyses. Toshihiko Matsumoto drafted the manuscript, and Takashi Usami, Akiho Nishimura, Sayako Higuchi, Kyoji Okita, and Takuya Shimane supervised manuscript preparation. All authors have reviewed, revised, and approved the final version of the manuscript for publication.

## CONFLICT OF INTEREST STATEMENT

The authors declare no conflicts of interest.

## ETHICS APPROVAL STATEMENT

This study was conducted with the approval of the Ethics Committee of the National Center of Neurology and Psychiatry (Approval No. A2024‐041).

## PATIENT CONSENT STATEMENT

The psychiatrists in charge at the participating institutions collected information by extracting anonymized clinical data from patients' medical records and transcribing it. During the survey period, the survey was announced, and potential participants were given the opportunity to opt out.

## CLINICAL TRIAL REGISTRATION

N/A.

## Data Availability

The data that support the findings of this study are available from the corresponding author upon reasonable request. The data supporting the findings of this study are not publicly available. This is because the ethics committee that approved this study did not approve the disclosure of data. If you wish to use the raw data, please contact the corresponding author of this paper. However, before actual use, you must obtain approval for the secondary use of the data from the ethics committee of the corresponding author's institution.
